# Impact of ethanol sclerotherapy on pelvic pain and quality of life in patients with endometriomas (ESCOMA study): protocol for a randomised clinical trial

**DOI:** 10.3389/frph.2026.1911200

**Published:** 2026-09-10

**Authors:** Rodrigo Guevara-Peralta, Jose Manuel Martinez-Garcia, Carlos Ortega-Expósito, Maria Jesús Pla-Farnós, Carlos Yeste, Judit Peñafiel, Júlia Guevara-Figueras, Jordi Ponce, Amparo García-Tejedor

**Affiliations:** 1 Department of Gynecology, Hospital Universitari Bellvitge, Universitat de Barcelona, Hospitalet de Llobregat, Barcelona, Spain; 2 Department of Gynecology, Hospital de Viladecans, Viladecans, Barcelona, Spain; 3 Department de Ciències Clíniques, Facultat de Medicina, Universitat de Barcelona, Hospitalet de Llobregat, Barcelona, Spain; 4 IDIBELL, Instituto de Investigación Biomédica de Bellvitge, Hospitalet de Llobregat, Barcelona, Spain

**Keywords:** endometrioma, endometriosis, ethanol sclerotherapy, fertility, ovarian reserve, pelvic pain, quality of life, randomised controlled trial

## Abstract

**Introduction:**

Endometriosis is a chronic gynecological disease associated with pelvic pain, infertility, and reduced quality of life. Ovarian endometrioma is its most common manifestation. Although laparoscopic cystectomy is widely used, concerns regarding ovarian reserve have led to increasing interest in conservative approaches. Ultrasound-guided ethanol sclerotherapy (EST) has emerged as a minimally invasive alternative with potential benefits for symptom control and reproductive outcomes. The ESCOMA trial aims to evaluate the impact of EST compared with expectant management on quality of life in women with ovarian endometriomas.

**Methods and analysis:**

ESCOMA is a prospective, multicentre, randomised controlled trial comparing ultrasound-guided ethanol sclerotherapy with expectant management. A total of 288 women with ovarian endometriomas will be randomised in a 1:1 ratio. The primary outcome is change in quality of life at 6 months, assessed using the Endometriosis Health Profile-5 (EHP-5). Secondary outcomes include pain reduction, changes in pain-related biomarkers, ovarian reserve markers, cyst size reduction, reproductive outcomes, adverse events and complications, diagnostic accuracy, and healthcare costs. Analyses will be performed according to both intention-to-treat and per-protocol principles.

**Ethics and dissemination:**

The study was approved by the Clinical Research Ethics Committee of Hospital Universitari de Bellvitge (PR070/25). Written informed consent will be obtained from all participants.

**Clinical Trial Registration:**

ClinicalTrials.gov, identifier NCT06955221.

## Strengths and limitations of this study

- First multicentre randomised clinical trial directly comparing ethanol sclerotherapy and expectant management for ovarian endometriomas.
- Primary outcome focused on quality of life, assessed with a validated and widely used tool (EHP-5).
- Inclusion of ovarian reserve markers and reproductive outcomes (pregnancy rates and time to conception) enhances the clinical relevance of the study; however, fertility outcomes will only be evaluated in the subgroup of women who express a desire for pregnancy, which reduces the sample size for these endpoints and may limit the statistical power to detect differences between groups.
- The additional assessment of healthcare costs provides valuable information on the health-economic impact of both treatment strategies.
- Although an open-label design may influence subjective outcomes such as quality of life, objective biomarkers and pregnancy outcomes are less susceptible to bias.

## Administrative information

Protocol version: v2.

## Sponsor and roles

- **Sponsor**: IDIBELL
- **Contact**: Dra Amparo García-Tejedor (agarciat@bellvitgehospital.cat).
- **Funder**: Instituto de Salud Carlos III (FIS PI24/01014).
- **Responsibilities**: The sponsor is responsible for trial conduct, regulatory obligations, and insurance. The funder has no role in study design, data collection, analysis, interpretation, manuscript writing, or decision to publish.
- **Committees**: Trial management is overseen by a Trial Steering Committee (investigators and biostatistician) and an independent Data Safety Monitoring Board (DSMB) every 6 months.

## Introduction

Endometriosis is a chronic, inflammatory, estrogen-dependent gynecological disease that affects approximately 5%–10% of women of reproductive age (1, 2) and is associated with infertility in up to 40% of cases (3). Ovarian endometrioma is its most frequent manifestation, occurring in 15%–45% of affected women (4). In addition to infertility, chronic pelvic pain is one of the main clinical symptoms, often causing work absenteeism and a substantial impairment in quality of life (QoL) (1, 2).

The optimal management of ovarian endometriomas remains controversial, and treatment should be individualized according to symptoms, reproductive plans, ovarian reserve, and cyst characteristics (1, 5). When surgical treatment is indicated, laparoscopic cystectomy remains the reference surgical technique because of its effectiveness in reducing pain and recurrence. However, surgery may reduce ovarian reserve owing to the inadvertent excision of healthy ovarian tissue, which is particularly detrimental in women with an already compromised reproductive potential (6, 7). Furthermore, postoperative recurrence rates remain high, ranging between 15%–30% (8). These limitations have stimulated increasing interest in conservative treatment strategies aimed at preserving ovarian reserve while achieving adequate symptom control, particularly in women wishing to conceive (1).

Current management of ovarian endometriomas has progressively shifted towards more conservative and fertility-preserving approaches, including expectant management, medical therapy, and minimally invasive interventions (1, 9). Treatment decisions should be individualized based on symptom severity, cyst characteristics, ovarian reserve, and the patient's reproductive goals (1, 9). Expectant management may be considered for selected asymptomatic women with ovarian endometriomas. However, because untreated endometriomas may be associated with a progressive decline in ovarian reserve, regular monitoring of cyst size and ovarian reserve is recommended (9). For symptomatic women who are not seeking immediate pregnancy, hormonal therapy is considered the first-line conservative treatment and includes combined oral contraceptives, progestins, and gonadotropin-releasing hormone (GnRH) agonists with add-back therapy (1, 10). Combined oral contraceptives are widely used as first-line medical therapy for pain and symptom control in women with endometriosis. However, evidence regarding their effect on endometrioma size and ovarian reserve remains limited (1, 9–11). Among progestins, dienogest is currently considered the first-line long-term medical treatment for endometriosis because of its effectiveness in reducing pain and improving endometriosis-related symptoms, with increasing evidence supporting its role in the management of ovarian endometriomas (9, 11–14). GnRH agonists remain an effective option for pain and symptom control in selected women with endometriosis. In women who are not seeking immediate pregnancy after surgery, postoperative treatment has been associated with lower endometrioma recurrence rates. However, their long-term use is limited by hypoestrogenic adverse effects; and add-back therapy is recommended to improve tolerability and facilitate prolonged treatment (10, 15). Although these conservative approaches play a key role in the management of ovarian endometriomas, each has specific indications and limitations, and none provides a definitive treatment while preserving ovarian reserve in all patients (1, 9, 11). Therefore, there remains a need for fertility-preserving alternatives that effectively control symptoms while minimizing the impact on ovarian reserve, particularly in women wishing to conceive (1, 9).

In this context, ultrasound-guided ethanol sclerotherapy has emerged as a promising minimally invasive, fertility-preserving alternative for selected women with ovarian endometriomas (16, 17). Its minimally invasive nature, outpatient setting, and potential to preserve ovarian reserve make ethanol sclerotherapy an attractive option for selected women with ovarian endometriomas, particularly those wishing to preserve their fertility (7, 18–20). Current evidence suggests that ethanol sclerotherapy achieves recurrence rates comparable to laparoscopic cystectomy while offering lower procedural costs and favorable reproductive outcomes, particularly in women with diminished ovarian reserve undergoing assisted reproductive treatment (16, 18, 21–23). However, despite these encouraging results, high-quality evidence on the long-term effects of ethanol sclerotherapy on quality of life, ovarian reserve, and reproductive outcomes remains limited (7, 16, 17, 23). These remaining uncertainties highlight the need for randomised clinical trials to better define the role of ethanol sclerotherapy in the management of ovarian endometriomas and provide the rationale for the present multicentre randomised clinical trial (16, 17, 23).

Beyond reproductive outcomes, improving quality of life is a key objective in the management of endometriosis. The Endometriosis Health Profile (EHP-30 and its shorter version, the EHP-5), developed at the University of Oxford, is a validated and widely used patient-reported outcome measure for assessing the impact of endometriosis on quality of life (24, 25).

Accordingly, the ESCOMA trial aims to compare ultrasound-guided ethanol sclerotherapy with expectant management in women with ovarian endometriomas, with the primary objective of comparing quality of life. Secondary objectives include assessing pain, ovarian reserve, reproductive outcomes, the need for subsequent surgery, safety, and treatment-related healthcare costs.

## Objectives

### Primary objective

- To compare the impact of ethanol sclerotherapy vs. expectant management on quality of life in women with ovarian endometriomas, assessed with the EHP-5 questionnaire at baseline and at 6 months.

### Secondary objectives

- To compare pain reduction between groups, measured by the Visual Analogue Scale (VAS) at 6 months and time to improvement.
- To assess changes in pain-associated biomarkers in serum [cortisol, C-reactive protein (CRP), interleukin-6 (IL-6)] and urine (cortisol, adrenaline, noradrenaline) between baseline and 6 months.
- To evaluate changes in ovarian reserve markers, including serum Anti-Müllerian Hormone (AMH) and antral follicle count (AFC), between baseline and 6 months.
- To compare the reduction in cyst size (largest diameter) between baseline and 6 months.
- To assess reproductive outcomes among women with pregnancy desire during the 24-month follow-up, including spontaneous pregnancies, assisted reproductive technology (ART) pregnancies, number of ART cycles required, and oocyte yield.
- To compare the number and type of adverse events and complications between groups.
- To identify the number of misdiagnosed cysts (non-endometriomas) in the sclerotherapy group.
- To compare treatment-related healthcare costs between groups.
- To assess endometrioma recurrence or progression during the 24-month follow-up.

## Methods and analysis

### Study design

This is a multicentre, open-label, randomised controlled clinical trial conducted across 39 hospitals in Spain. Participants will be randomised in a 1:1 ratio to undergo EST or expectant management. Given the nature of the intervention, blinding is not feasible; however, objective outcomes will mitigate bias.

#### Concomitant care

Both groups may receive complementary medical treatment at the discretion of the treating specialist, in accordance with the clinical protocols of each participating centre. In most cases, such treatments will already have been prescribed before study entry.

### Participants

Eligibility criteria are listed below

#### Inclusion Criteria

- Female sex
- Age ≥18 and ≤45 years
- Ultrasound suspicion of unilocular endometrioma or with a thin septum less than 3 mm
- Size between 30 and 100 mm, persistent for at least 3–6 months since diagnosis
- Ca125 marker < 300 UI/mL and HE4 < 70 pM
- Bilateral endometriomas.
- Recurrent endometriomas
- Previous ovarian intervention, including ovarian surgery or sclerotherapy
- Signed informed consent

#### Exclusion Criteria

- Age <18 or >45 years
- History of ovarian or uterine cancer
- Endometrioma size < 30 mm or >100 mm
- Indication for surgical treatment of the endometrioma due to suspected severe extra-ovarian endometriosis or any other cause. In cases of clinical suspicion of severe extra-ovarian endometriosis, pelvic MRI will be performed to assess disease extent and confirm eligibility.
- Ultrasound suspicion of dermoid cysts, anechoic cysts, or cysts with high risk of malignancy
- Ca125 > 300 UI/mL
- HE4 > 70 pM
- Pregnant women
- Patients who do not wish to participate in the study or who are mentally incapacitated

All participant centers are members of the ESCOMA team. The list of participating Hospitals is shown in the Supplementary Appendix S1.

### Interventions

Intervention group: Ultrasound-guided ethanol sclerotherapy (EST).

#### Procedure

- Analgesic treatment is administered before the procedure, consisting of ibuprofen 1 h before and diazepam ½ hour before. On the day of the puncture, the patient must not present purulent leucorrhoea or genital bleeding.
- A single puncture is performed, preferably via the vaginal route, although an abdominal approach may be used if required by the patient's condition or by the cyst's characteristics, for example in virginal patients or when marked vascularization is observed along the puncture pathway.
- The abdominal wall or vaginal canal is disinfected with a povidone–iodine solution.
- A sterile 17-gauge needle (BD Medical Franklin Lakes, NJ, Becton, Dickinson and Company) is used. Under ultrasound guidance, the needle is advanced into the centre of the cyst and connected, through an extension tube and a three-way stopcock, to a vacuum aspiration system.
- Aspiration of the endometrioma contents may require dilution with saline solution (SS) when the fluid is thick. The aspirated fluid is measured using a syringe or an adapted measurement system.
- After nearly complete aspiration, several washes with Saline solution (SS) are performed until clear fluid is obtained.
- A volume of 100% ethanol equivalent to two-thirds of the aspirated volume is then instilled into the cyst, without exceeding 100 mL.
- The ethanol is left in place for 15 min without moving the needle, which remains inside the cyst.
- Subsequently, the cyst is emptied and washed twice with SS, using the same volume as the instilled ethanol.
- The aspirated fluid is sent to the pathology department for cytological examination to confirm the absence of atypical cells.
- The 100% ethanol solution used at all participating centres will be identical. The product will be supplied by *Farmacia Carreras*, the authorized provider for hospitals throughout Spain, ensuring consistency and avoiding potential bias related to variations in preparation.

#### Expectant management group

Participants will continue the treatment they were receiving before study inclusion, or no treatment if none was previously prescribed. During follow-up, medical treatment may be initiated or modified according to clinical circumstances, patient preference, and usual clinical practice. Recommendations are provided to investigators to promote consistency in the management of the expectant-management group across participating centres (Supplementary Appendix 3); however, treatment decisions are not mandated by the study protocol. All concomitant treatments and subsequent changes will be prospectively recorded in the study database. Analgesia and supportive care are permitted in both groups. To minimize potential imbalance in this relevant clinical factor, randomisation will be stratified according to the presence or absence of concomitant hormonal treatment at study inclusion. In addition, the potential effect of hormonal treatment received during follow-up will be accounted for in the statistical analysis, including appropriate adjusted and sensitivity analyses.

### Outcomes

Primary outcome: Quality of life assessed with the EHP-5 questionnaire, a validated self-administered patient-reported outcome measure, at baseline and 6 months.

#### Secondary outcomes

- Pain reduction (VAS score at 6 months and time to improvement)
- Changes in pain-associated biomarkers in serum (cortisol, CRP, IL-6) and urine (cortisol, adrenaline, noradrenaline)
- Ovarian reserve markers (AMH and AFC)
- Reduction in cyst size (largest diameter)
- Reproductive outcomes (spontaneous pregnancies, ART pregnancies, number of ART cycles, oocyte yield),
- Adverse events and complications,
- Number of misdiagnosed cysts (non-endometrioma)
- Treatment-related direct medical costs from the healthcare system perspective.
- Endometrioma recurrence or progression.

Long-term outcomes assessed during follow-up up to 24 months will include endometrioma recurrence or progression, subsequent interventions, and reproductive outcomes among women with pregnancy desire, including spontaneous pregnancies and ART-related outcomes (number of ART cycles, number of oocytes retrieved, and pregnancies achieved through ART).

A detailed description of all secondary outcomes, including definitions, timing and methods of aggregation, is provided in Supplementary Table S1.

### Sample size

For the primary endpoint (quality of life), the sample size was estimated based on published data reporting an improvement in quality of life—considering patient-reported pain—of approximately 55.25% (range 53%–60%) after conservative management and 71.5% (range 63%–80%) after EST. Assuming a two-sided *α* of 0.05 and a statistical power greater than 0.80, 144 participants per group are required to detect a statistically significant difference between the two proportions (p_1_ = 0.5525 and p_2_ = 0.715). Considering a 5% loss to follow-up, the total sample size will be 288 participants (144 per group). Calculations were performed using the arcsine (arcsin–square-root) approximation for comparison of proportions.

For the fertility substudy (subgroup of women expressing a desire for pregnancy), published data indicate pregnancy rates of approximately 48% after EST (range 37%–55%) and 15% with conservative management. Assuming a two-sided *α* of 0.05 and a *β* of 0.20 (power = 0.80), 33 participants per group are required to detect this difference (p_1_ = 0.48, p_2_ = 0.15). Allowing for a 10% loss to follow-up, the final target will be 37 participants per group, yielding a total of 74 women for the fertility analysis.

These calculations ensure adequate statistical power to detect clinically meaningful differences in both quality of life and reproductive outcomes between treatment groups.

### Randomisation and allocation

Randomisation will be performed by an independent statistician using computer-generated permuted blocks, stratified by prior ovarian intervention (surgery or EST), pregnancy desire, and hormonal treatment. Allocation will be centrally managed through a secure, web-based REDCap platform with concealed randomisation sequence. Site investigators will enrol eligible participants, and group allocation will be automatically assigned by the electronic system.

### Blinding

This is an open-label, non-blinded study. Both physician and patient will be aware of the allocated treatment arm, since blinding is not considered feasible when comparing an interventional procedure (EST) with expectant management according to standard clinical practice. The absence of blinding may influence patient-reported quality of life outcomes; however, it should not affect reproductive or biomarker outcomes, which are objective measures.

### Statistical analysis

Primary analysis will use mixed linear regression models with clustering by patient to compare QoL scores. Secondary analyses will include chi-square for categorical outcomes, repeated measures ANOVA, Kaplan–Meier survival curves for pregnancy rates, and Mann–Whitney U tests for oocyte counts. Analyses will be performed on both intention-to-treat and per-protocol populations. A statistical analysis plan will be finalised prior to database lock.

Secondary outcomes will be analysed as defined in Supplementary Table S1. Continuous outcomes (e.g., VAS, AMH, AFC, cyst diameter, oocyte yield, biomarker levels) will be compared between groups using appropriate parametric or non-parametric tests depending on distribution. Categorical outcomes (e.g., pregnancy rates, adverse events, misdiagnosis) will be compared using *χ*^2^ or Fisher's exact test. Time-to-event outcomes (e.g., time to clinical improvement, time to spontaneous pregnancy) will be analysed using Kaplan–Meier curves and log-rank tests. Effect estimates will be presented with 95% confidence intervals. A detailed Statistical Analysis Plan is provided in the Supplementary Appendix S7.

#### Handling of concomitant treatments

All concomitant medical treatments will be recorded at baseline and during follow-up. Sensitivity analyses will be performed to explore whether concomitant medication use modifies the treatment effect on primary and secondary outcomes. These analyses will be exploratory in nature.

### Data management and monitoring

Data will be entered into a secure REDCap database hosted at IDIBELL. Monitoring will be performed by an independent contract research organisation (CRO) hosted in IDIBELL, including initiation, interim, and close-out visits. A Data Safety Monitoring Board (DSMB) will meet at 6, 12, and 18 months to review safety and efficacy. Details are provided in the Supplementary Appendix.

### Ethics and dissemination

The trial complies with the Declaration of Helsinki and Good Clinical Practice. Ethics approval has been obtained from the Bellvitge University Hospital Research Ethics Committee (PR 070/25). Written informed consent will be obtained from all participants. Data will be pseudonymised and stored securely. Results will be disseminated through peer-reviewed journals, conferences, professional networks, and patient associations. Regardless of outcomes, results will be published.

### Patient and public involvement

Patients and the public were not involved in the design or conduct of the study. Dissemination will be facilitated through patient associations, public talks, and online platforms.

## Discussion

This protocol describes the first multicentre randomised controlled trial designed to compare ultrasound-guided ethanol sclerotherapy (EST) with expectant management in women with ovarian endometriomas. Although laparoscopic cystectomy remains the most widely used treatment, increasing concerns regarding its impact on ovarian reserve have led to growing interest in less invasive alternatives. EST has shown promising results in observational studies and small comparative cohorts, but high-quality randomised evidence remains limited. The ESCOMA trial aims to address this knowledge gap by providing robust data from a large multicentre population representative of routine clinical practice.

A major strength of this study is the selection of quality of life as the primary outcome. Endometriosis is a chronic disease with a substantial physical, psychological, and social burden, and treatment success should not be evaluated solely through anatomical or reproductive outcomes. The use of the EHP-5 questionnaire, a validated disease-specific instrument, allows the assessment of outcomes that are directly meaningful to patients and aligns with current recommendations promoting patient-centred care in endometriosis research.

Another important aspect of this trial is the comprehensive evaluation of secondary outcomes. In addition to pain relief and cyst size reduction, the study incorporates ovarian reserve markers, reproductive outcomes, pain-related biomarkers, treatment-related complications, diagnostic accuracy, and healthcare costs. This multidimensional approach will provide a broader understanding of the potential benefits and limitations of EST and may help clinicians individualize treatment decisions according to patient priorities, particularly among women wishing to preserve fertility.

The inclusion of ovarian reserve assessment is particularly relevant. Surgical treatment of endometriomas has consistently been associated with reductions in serum anti-Müllerian hormone levels and potential damage to healthy ovarian tissue. As many affected women are diagnosed during their reproductive years, preserving ovarian function has become a key consideration when selecting therapeutic strategies. If EST demonstrates comparable symptom control while minimizing the impact on ovarian reserve, it may represent an attractive alternative to surgery in selected patients.

The economic evaluation of the ESCOMA study will compare direct medical costs between treatment groups, including healthcare resource utilization and the management of treatment-related complications. This will allow us to assess the healthcare costs associated with EST compared with expectant management.

Some limitations should be considered when interpreting the results of this study. First, the open-label design may influence patient-reported outcomes such as quality of life and pain perception. However, blinding is not feasible when comparing an invasive procedure with expectant management, and several secondary outcomes, including biomarkers, ovarian reserve parameters, and reproductive outcomes, are objective measures less susceptible to observer bias. Second, variations in clinical practice among participating centres may introduce heterogeneity. Nevertheless, the multicentre nature of the study enhances the external validity and generalizability of the findings. Third, reproductive outcomes will be evaluated only among women who express a desire for pregnancy, reducing the effective sample size for fertility-related analyses. Finally, although the primary endpoint will be assessed at 6 months, patients will be followed for a total of 24 months, allowing longer-term assessment of endometrioma recurrence or progression, reproductive outcomes, and subsequent interventions.

In conclusion, the ESCOMA trial has the potential to generate high-quality evidence regarding the role of EST in the management of ovarian endometriomas. By integrating patient-reported outcomes, reproductive outcomes, biological markers, and economic analyses, this study may contribute to redefining treatment algorithms and informing future clinical guidelines for women affected by this common and challenging condition.

## Data sharing

De-identified participant data and statistical code will be available upon reasonable request after publication, subject to ethics approval and GDPR compliance. Requests should be directed to the corresponding author.

## Post-trial care

The intervention is minimally invasive and part of standard practice; any harm will be managed per institutional policies. No additional post-trial care is planned.

## Authorship and dissemination

Authorship will follow ICMJE criteria. Results will be submitted within 12 months after database lock and posted on the trial registry. The full protocol and SAP will be publicly accessible. Findings will also be disseminated to participants, healthcare professionals, and policy makers through open-access publication and presentations at international conferences.
