# Supplementary material for: Impact of ethanol sclerotherapy on pelvic pain and quality of life in patients with endometriomas (ESCOMA study): protocol for a randomised clinical trial
Source: Front Reprod Health. 2026 Sep 10;8:1911200. doi: 10.3389/frph.2026.1911200 (PMC13601309; doi:10.3389/frph.2026.1911200)
Supplement: Supplementary file 1 [file Datasheet1.pdf]

ESCOMA – Supplementary Material Structure

Supplementary Figure S1

SPIRIT schedule of enrolment, interventions, and assessments.

Timepoint	Enrolment (Screening)	Allocation (Baseline)	Procedure (Intervention)	6 months	24 months	Follow-up
Eligibility screen	X					
Informed consent	X					
Randomisation		X				
Ethanol sclerotherapy / Expectant management			X			
EHP-5 QoL questionnaire		X		X		
VAS pain score		X		X		
Transvaginal ultrasound (AFC, cyst size)		X		X	X	
Serum AMH		X		X		
Biomarkers (blood/urine: cortisol, CRP, IL-6, adrenaline, noradrenaline)		X		X		
Adverse events			X	X	X	X
Pregnancy outcomes (spontaneous, ART)				X	X	X
Endometrioma recurrence or progression				X	X	X
Healthcare costs		X		X	X	X

Supplementary Table S1

Detailed description of secondary outcomes, definitions, timing of assessment, and methods of aggregation.

**Secondary outcome**	**Measure / definition**	**Time point(s)**	**Method of aggregation**
Clinical improvement (pain)	Change in VAS score; time to improvement	Baseline, 6 months	Mean ± SD; median time (IQR)
Pain-associated biomarkers	Blood: cortisol, CRP, IL-6;<br><br>Urine: cortisol, adrenaline, noradrenaline	Baseline, 6 months	Mean ± SD or median (IQR)
Spontaneous pregnancies	Number of pregnancies; time to conception	Up to 24 months	Count; Kaplan–Meier curves
ART pregnancies	Number of pregnancies via ART; cycles required	Up to 24 months	Count; mean cycles
Oocyte yield in ART	Number of oocytes retrieved	During ART cycles	Mean ± SD
Antral follicle count (AFC)	AFC by transvaginal ultrasound	Baseline and 6 months	Mean ± SD
Anti-Müllerian Hormone (AMH)	Serum AMH (ng/mL) change from baseline	Baseline and 6 months	Mean ± SD
Cyst size reduction	Change in mean cyst diameter (mm)	Baseline, 6 and 24 months	Mean ± SD
Adverse events and complications	Clavien Dindo	Throughout study	Count, %
Misdiagnosed cysts	Number of patients with alternative diagnosis	During procedure	Count
Healthcare costs	Direct medical costs associated with treatment, including	Baseline, 6 and 24 months	Total and mean direct medical costs per

	management of treatment-related complications		patient and treatment group
Endometrioma recurrence or progression	Recurrence of an ovarian endometrioma following EST or progression of the existing endometrioma in the expectant management group, assessed by transvaginal ultrasound	Up to 24 months	Number and proportion of patients with recurrence/progression; time to recurrence/progression, where applicable

Supplementary Appendix 1

Participating centres and ESCOMA Study Group investigators.

1. Hospital Universitario de Bellvitge
2. Hospital de Viladecans
3. Consorci Sanitari de l'Anoia (Igualada)
4. Hospital Universitario Central de Asturias
5. Hospital Universitario Son Espases
6. Hospital del Mar
7. Hospital Clínico Universitario Lozano Blesa
8. Parc Sanitari Sant Joan de Deu (Sant Boi)
9. Consorci Corporació Sanitària Parc Taulí
10. Hospital Universitario 12 de Octubre
11. Hospital de la Santa Creu i Sant Pau
12. Hospital Universitario de Donostia
13. Hospital Universitario Ramón y Cajal
14. Hospital Universitario de Álava - Txagorritxu
15. Hospital Recoletas Salud Campo Grande
16. Hospital Universitario Germans Trias i Pujol (Can Ruti)
17. Hospital Universitari General de Catalunya
18. Consorci Sanitari Integral
19. Hospital Mutua Terrassa
20. Hospital Universitario Joan XXIII
21. Virgen Arrixaca Murcia
22. Fundación Althaia Manresa
23. Ginefiv-GeneraLife
24. Consorci Sanitari de Terrassa
25. QuironSalud Málaga
26. Virgen Macarena Sevilla
27. Hospital General Universitario Los Arcos del Mar Menor
28. Hospital Universitario San Pedro

29. Hospital de Santa Caterina
30. Hospital General de Granollers
31. Hospital Universitario Doctor José Molina Orosa
32. Hospital Infanta Cristina
33. Hospital Universitario de Jaén
34. Hospital Universitario de Burgos
35. Hospital General de Valencia
36. Hospital San Juan de Dios del Aljarafe
37. Hospital Universitario de Cabueñes
38. Hospital Clínico San Carlos
39. Hospital Vithas Xanit Internacional

Supplementary Appendix 2

Standardized ethanol sclerotherapy procedure (SOP): patient preparation, analgesia protocol, ultrasound-guided puncture technique, ethanol instillation procedure, cytological sample handling, and post-procedure follow-up.

Procedure: Ultrasound-guided Ethanol Sclerotherapy

The procedure is performed on an outpatient basis, either transvaginally or transabdominally depending on cyst location. After disinfecting the vaginal canal or abdominal wall with an iodine solution, a fine needle is introduced under ultrasound guidance to aspirate the cyst contents. When necessary, the fluid may be diluted with saline solution to facilitate aspiration. After nearly complete evacuation, the cyst is washed with saline and a small volume of sterile alcohol is injected through the same needle. The alcohol remains inside the cyst for about 15 minutes while you remain lying on the examination couch. The cyst is then emptied and flushed with saline to remove any remaining alcohol.

The procedure is performed under ambulatory surgery conditions. In the absence of complications, you will be discharged approximately one hour after completion. Total hospital stay is expected to be between 1½ and 2 hours. You may resume normal activities the following day. In case of discomfort, standard analgesics (except aspirin and derivatives) may be used.

The total Follow-up follow-up period will be twenty-four months.

Supplementary Appendix 3 –

Recommended Medical Management Algorithm for Patients allocated to Expectant Management

To reduce heterogeneity in concomitant medical treatment across participating centres while preserving the pragmatic nature of the expectant-management arm, investigators are provided with the following recommendations for medical management.

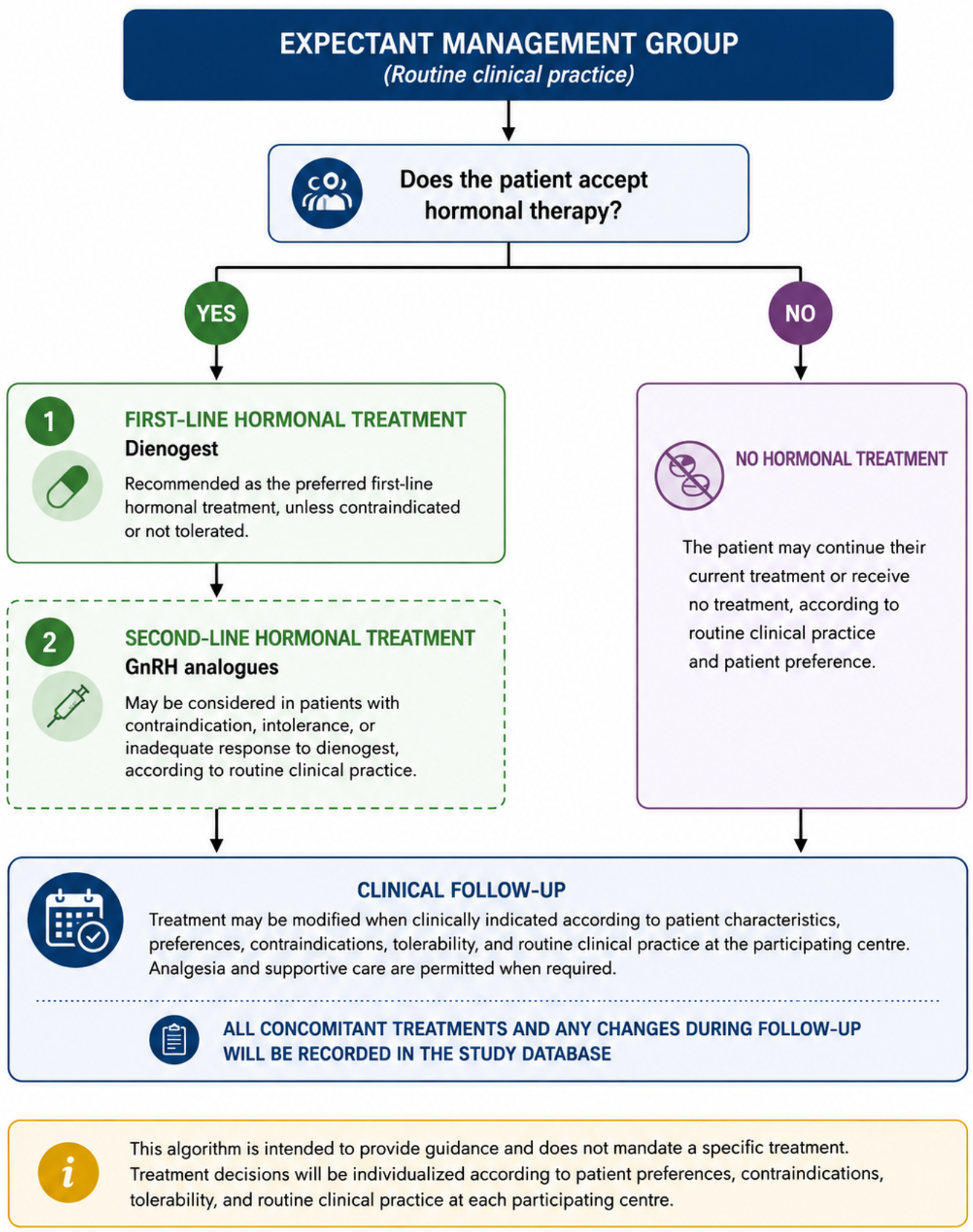

Figure S1. Suggested treatment algorithm for the expectant management group.

Supplementary Appendix 4
Intervention modification, adherence monitoring, and concomitant treatments.

Criteria for modification/discontinuation: procedure will be aborted in case of uncontrolled bleeding, suspected malignancy, or patient intolerance. Adherence/fidelity: operators follow a standardized SOP; fidelity is checked by procedure checklist and audit of 10% random cases.

Concomitant care: Concomitant medical treatments will be permitted in both study arms, according to local clinical practice and the treating specialist's judgement. Most participants were already receiving such treatments prior to enrolment.

Supplementary Appendix 5
Adverse event reporting and safety monitoring, including Clavien–Dindo classification, SAE reporting, and DSMB responsibilities.

Adverse events are collected at all visits, graded by Clavien-Dindo classification by staff not involved in the intervention. Serious adverse events are reported to the sponsor and ethics committee within 24–72h.

Supplementary Appendix 6
Site and operator eligibility requirements.

Site eligibility: Public hospitals with a gynaecology department and access to transvaginal ultrasound and outpatient procedural facilities. Participating centres must be able to perform ultrasound-guided puncture and follow-up according to the study protocol.

Operator eligibility: The procedure will be performed by gynaecologists trained in ultrasound-guided cyst puncture and familiar with the study protocol. Sites with limited prior experience will receive specific practical training and supervision before patient recruitment.

Supplementary Appendix 7

Statistical Analysis Plan (SAP): primary and secondary analyses, missing data handling, sensitivity analyses, subgroup analyses, and health economic analysis.

Primary outcome: Change in EHP-5 score from baseline to 6 months will be analysed using mixed-effects models for repeated measures, adjusting for baseline values and stratification factors. Analyses will follow the intention-to-treat principle.

Secondary outcomes will be analysed as defined in Supplementary Table S1. Continuous variables will be compared using parametric or non-parametric tests depending on distribution. Categorical outcomes will be compared using χ^2 or Fisher's exact test. Time-to-event outcomes will be analysed using Kaplan–Meier curves and log-rank tests. Effect estimates will be presented with 95% confidence intervals.

Ovarian reserve analysis: Changes in AMH will be assessed at the individual level by comparing baseline and follow-up values for each participant. Baseline age and AMH levels will be described by treatment group. The relationship between changes in AMH and relevant clinical variables, including age, parity, and concomitant hormonal treatment, will also be explored.

Stratification and subgroup analyses: Randomization will be stratified by previous ovarian intervention (surgery or sclerotherapy), pregnancy desire, and concomitant hormonal treatment. Bilateral endometriomas will not be included as an additional stratification factor. Their potential influence on study outcomes will be assessed in a subsequent sensitivity/subgroup analysis.

Concomitant treatments:

Concomitant medical treatments will be documented at baseline and throughout follow-up, including any initiation or modification of treatment. Predefined subgroup analyses according to concomitant hormonal treatment will be performed to assess its potential influence on primary and secondary outcomes. Exploratory sensitivity analyses will additionally assess whether concomitant treatment modifies the effect of the intervention

Health economic analysis:

The economic analysis will be conducted from the healthcare system perspective and will include direct medical costs associated with each treatment strategy. Cost analysis will include healthcare staff, procedure-related resources and supplies, medications, inpatient services when required, management of treatment-related complications, and length of hospital stay. Indirect costs, including productivity loss, work absenteeism, and transportation costs, will not be included.

Supplementary Appendix 8

Participant Information Sheet and Informed Consent Form (PIS/ICF).

A model informed consent form is included in this supplement. It describes study purpose, procedures, risks, benefits, confidentiality, and voluntary participation. The Patient Information Sheet and Informed Consent are shown below. The official CEIC-approved version with institutional logos will be provided as Supplementary File 2 if required.

Participant Information Sheet and Informed Consent Form (PIS/ICF)

Study title: Impact of Alcohol Sclerotherapy on Pelvic Pain and Quality of Life in Patients with Ovarian Endometriomas (ESCOMA study)

Participant Information Sheet

You have been diagnosed with an ovarian cyst compatible with an endometrioma (an ovarian cyst formed by tissue similar to the endometrium, which can cause pain and affect fertility). The currently recommended treatment is medical management. However, the presence of an endometrioma can cause chronic pelvic pain and may reduce fertility rates.

We invite you to participate voluntarily in a clinical study comparing the benefits of a minimally invasive procedure called puncture and alcohol sclerotherapy of the endometrioma, with expectant management (medical observation without surgical intervention, with periodic follow-up scans). Alcohol sclerotherapy is a simple procedure already used in some hospitals, but its potential advantages over expectant management in terms of symptom improvement and fertility preservation still need to be validated.

To properly assess the advantages and disadvantages of these two therapeutic approaches, it is necessary to compare groups of patients with similar characteristics undergoing different treatments. The type of treatment you will receive will be determined randomly by a computer programme (ratio 1:1) once you have agreed to participate. Neither you nor your doctor will be able to choose which treatment group you are assigned to.

Your symptoms will be evaluated at baseline and at six months after the procedure, including pain using a 1–10 visual analogue scale (VAS) and a quality-of-life questionnaire (EHP-5). Blood tests will measure Anti-Müllerian Hormone (AMH) at baseline and six months to assess fertility potential.

Follow-up will be carried out six months after the procedure, in a manner similar to standard clinical practice, and will not require any additional visits apart from blood tests. The total follow-up period will last between six months and two years, depending on your clinical situation.

Procedure: Puncture and Ethanol Sclerotherapy

The procedure is performed on an outpatient basis, either transvaginally or transabdominally depending on cyst location. After disinfecting the vaginal canal or abdominal wall with an iodine solution, a fine needle is introduced under ultrasound guidance to aspirate the cyst contents. When necessary, the fluid may be diluted with saline solution to facilitate aspiration. After nearly complete evacuation, the cyst is washed with saline and a small volume of sterile alcohol is injected through the same needle. The alcohol remains inside the cyst for about 15 minutes while you remain lying on the examination couch. The cyst is then emptied and flushed with saline to remove any remaining alcohol.

The procedure is performed under ambulatory surgery conditions. In the absence of complications, you will be discharged approximately one hour after completion. Total hospital stay is expected to be between 1½ and 2 hours. You may resume normal activities the following day. In case of discomfort, standard analgesics (except aspirin and derivatives) may be used.

Risks and possible complications

The usual treatment for endometriomas consists of observation with periodic ultrasound monitoring or surgery when symptoms or cyst size warrant it. This study aims to compare the efficacy and safety of puncture and alcohol sclerotherapy with observation, evaluating improvements in pain and fertility preservation.

Specific complications of the procedure may include mild abdominal discomfort similar to menstrual pain, dizziness, or light vaginal bleeding that resolves spontaneously. Rarely, heavier bleeding or pelvic infection may occur, presenting as severe lower abdominal pain and occasionally requiring emergency surgery. Ethanol intoxication is extremely uncommon.

Other potential risks common to both groups include the inability to treat associated lesions and the absence of a complete histopathological study since the cyst is not excised. However, preoperative imaging and laboratory assessments allow for a highly reliable diagnosis of benignity, with the risk of underlying malignancy being less than 1%.

Anaesthesia: The procedure is generally well tolerated and does not require anaesthesia. To minimise discomfort, participants are instructed to take ibuprofen orally and diazepam sublingually one hour before the procedure.

Pathology: The aspirated material will be sent for cytological examination to confirm benignity. Participants will be informed of the results by phone.

Potential benefits: This study aims to demonstrate that puncture and alcohol sclerotherapy of ovarian endometriomas helps to reduce pain and improve quality of life. We also expect that markers of ovarian reserve and fertility potential will remain stable across both treatment groups. Information obtained may contribute to improving the treatment of women with the same condition. However, participation in this study may not provide additional benefit beyond standard clinical care.

Confidentiality:

By signing this document, you consent to participate in this study and to the processing of your clinical and personal data for study purposes. Data processing will comply with EU Regulation (GDPR 2016/679) and Spanish Law 3/2018 on data protection. Your information will be stored in a coded database, meaning that identifiable information will be replaced with an alphanumeric code. Only the research team, monitors, the Ethics Committee, and regulatory authorities will have access to your records.

You have the right to access, rectify, limit, or delete your personal data, and to request a copy or transfer of your data to a third party (data portability). These rights can be exercised through the Principal Investigator or the institution's Data Protection Officer. Data will be retained for a minimum of 25 years as required by clinical research regulations.

`Voluntary participation and right to withdraw

Participation in this study is voluntary. If you choose not to participate, you will continue to receive standard medical care. You may withdraw from the study at any time without providing a reason and without any impact on your ongoing medical treatment. The study has been reviewed and approved by the Clinical Research Ethics Committee of your centre.

Informed Consent Form

Study title: Impact of Alcohol Sclerotherapy on Pelvic Pain and Quality of Life in Patients with Ovarian Endometriomas (ESCOMA study)
Version 3 – 09 October 2025
Principal Investigator: ____________________________
Department of Gynaecology – Hospital ____________________________

Participant name: ____

I have read and understood the information sheet provided to me.
I have had the opportunity to ask questions about the study.
I have received satisfactory answers to my questions.
I have spoken with: ____ (name of investigator)

I understand that my participation is voluntary.
I understand that I can withdraw from the study:
– Whenever I wish
– Without giving reasons
– Without any effect on my medical care.

I freely agree to participate in this study and consent to the access and use of my data as described in the information sheet.

☐ Yes ☐ No

Participant signature: ____________________________ Date: ____________

Investigator signature: ____________________________ Date: ____________

This document will be signed in duplicate. One copy will remain with the investigator, and one will be given to the participant.

Supplementary Appendix 9

EHP-5 questionnaire used for quality-of-life assessment (used under licence from the University of Oxford).

DURANTE LAS ÚLTIMAS 4 SEMANAS,
¿CÓMO ES QUE, A CAUSA DE SU ENDOMETRIOSIS...?

		Nunca	Rarame nte	A veces	Frecuen temente	Siempre
**1.**	¿Le resultó dificil caminar debido al dolor?	☐	☐	☐	☐	☐
**2.**	¿Sientes que tus síntomas gobiernan tu vida?	☐	☐	☐	☐	☐
**3.**	¿Tuviste cambios de humor?	☐	☐	☐	☐	☐
**4.**	¿Sentiste que los demás no entienden por lo que estás pasando?	☐	☐	☐	☐	☐
**5.**	¿Sentiste que tu apariencia se ha visto afectada?	☐	☐	☐	☐	☐
